# Is onchocerciasis elimination in Africa feasible by 2025: a perspective based on lessons learnt from the African control programmes

**DOI:** 10.1186/s40249-018-0446-z

**Published:** 2018-07-03

**Authors:** Yankum Dadzie, Uche V. Amazigo, Boakye A. Boatin, Azodoga Sékétéli

**Affiliations:** 1 P.O. Box OS-1905, Accra, Ghana; 2 P. O. Box 3397, Main Post Office, Okpara Avenue, Enugu, Nigeria; 3 P. O. Box CT 1380, Accra, Ghana; 4 BP 3841, Lomé 01, Togo

**Keywords:** Onchocerciasis, Elimination, Vector control, Ivermectin, Conceptual framework

## Abstract

**Background:**

Onchocerciasis is found predominantly in Africa where large scale vector control started in 1974. Registration and donation of ivermectin by Merck & Co in 1987 enabled mass treatment with ivermectin in all endemic countries in Africa and the Americas. Although elimination of onchocerciasis with ivermectin was considered feasible only in the Americas, recently it has been shown possible in Africa too, necessitating fundamental changes in technical and operational approaches and procedures.

**Main body:**

The American programme(OEPA) operating in onchocerciasis epidemiological settings similar to the mild end of the complex epidemiology of onchocerciasis in Africa, has succeeded in eliminating onchocerciasis from 4 of its 6 endemic countries. This was achieved through biannual mass treatment with ivermectin of 85% of the eligible population, and monitoring and evaluation using serological tests in children and entomological tests.

The first African programme(OCP) had a head start of nearly two decades. It employed vector control and accumulated lots of knowledge on the dynamics of onchocerciasis elimination over a wide range of epidemiological settings in the vast expanse of its core area. OCP made extensive use of modelling and operationalised elimination indicators for entomological evaluation and epidemiological evaluation using skin snip procedures.

The successor African programme(APOC) employed mainly ivermectin treatment. Initially its objective was to control onchocerciasis as a public health problem but that objective was later expanded to include the elimination of onchocerciasis where feasible. Building on the experience with onchocerciasis elimination of the OCP, APOC has leveraged OCP’s vast modelling experience and has developed operational procedures and indicators for evaluating progress towards elimination and stopping ivermectin mass treatment of onchocerciasis in the complex African setting.

**Conclusions:**

Following the closure of APOC in 2015, implementation of onchocerciasis elimination in Africa appears to overlook all the experience that has been accumulated by the African programmes. It is employing predominantly American processes that were developed in a dissimilar setting from the complex African onchocerciasis setting. This is impeding progress towards decisions to stop intervention in many areas that have reached the elimination point. This article summarizes lessons learned in Africa and their importance for achieving elimination in Africa by 2025.

**Electronic supplementary material:**

The online version of this article (10.1186/s40249-018-0446-z) contains supplementary material, which is available to authorized users.

## Multilingual abstracts

Please see Additional file 1 for translations of the abstract into the six official working languages of the United Nations.

## Background

Onchocerciasis is one of two diseases among twenty covered by the Neglected Tropical Diseases Department of the World Health Organization (WHO) that has been identified for elimination by 2025 [1]. About 200 million people are at risk of onchocerciasis infection and more than 99% of the global disease burden is present in Africa [2, 3]. It has been estimated that before the start of large scale control 33 million people were infected [4, 5]. Large scale control of onchocerciasis started in 1974 with the creation of the Onchocerciasis Control Programme in West Africa (OCP) [6]. The OCP applied vector control to achieve its objective of eliminating onchocerciasis as a public health problem and as an obstacle to socio-economic development in the West African countries involved. Though vector control proved very successful in controlling onchocerciasis and even interrupting transmission in the core area of the OCP, the method could not be extended elsewhere because of the high cost of implementation as well as the topography of other areas which made access and aerial spraying of complex breeding sites technically difficult if not outright impossible. The registration in 1987 of ivermectin, a safe and effective microfilaricide suitable for mass treatment of onchocerciasis and given free of charge by the manufacturer, Merck & Co, led to the creation of new onchocerciasis control programmes to cover the rest of Africa outside the OCP through the African Programme for Onchocerciasis Control (APOC) and the Americas through the Onchocerciasis Elimination Programme for the Americas (OEPA) [7, 8].

To date, a lot of success has been achieved: in Africa the disease has been eliminated as a public health problem across the length and breadth of the continent [9, 10]. In America onchocerciasis has been eliminated in four of the six endemic countries [2]. However, it is worthy of note that, at the start of the century, an international conference on ‘eradicability’ of onchocerciasis by experts in various related fields, arrived at the conclusion that the use of ivermectin, a microfilaricide for mass treatment of populations, was unlikely to be able to eliminate onchocerciasis in Africa though it would be feasible to do so in the Americas, because of the complexity of its epidemiology in Africa compared with that in the Americas [11].

**Fig. 1:**
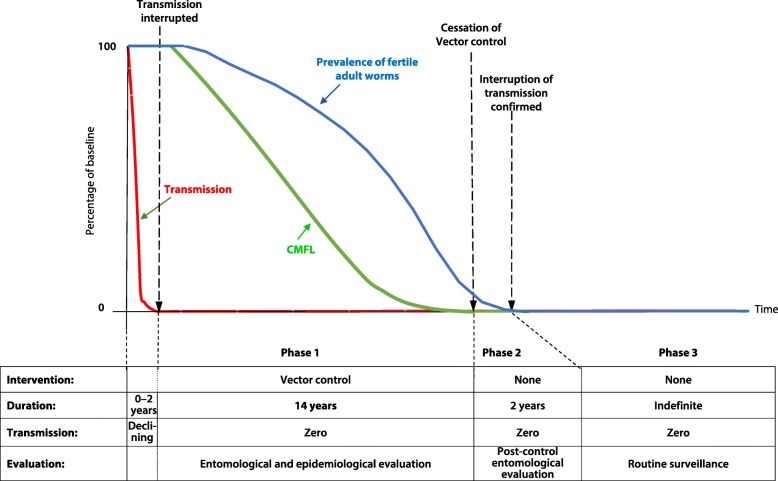
Conceptual Framework of elimination by vector control (OCP)

In Africa, the first programme, the OCP, initially carried out vector control. When ivermectin became available, the programme used it in combination with vector control in some areas while applying ivermectin alone in others [9]. As vector control focused on interrupting transmission, much was learnt about onchocerciasis transmission dynamics and the required duration of control. Extensive evaluation data combined with epidemiological modelling indicated that the reproductive lifespan of the adult female onchocercal worm was about 10 years and that, taking worm lifespan variability into account, 14 years of interruption of transmission were required to achieve elimination [12–14]. Figure 1 shows the conceptual framework for the vector control strategy of the OCP.

The second programme, APOC, used mass treatment with ivermectin as its principal intervention strategy, and applied Community-Directed Treatment with ivermectin (CDTi), as the method for ivermectin mass delivery. The operational implementation of CDTi in defined onchocerciasis endemic areas in countries were referred to as the CDTi projects. Though the CDTi method proved sustainable for long periods the question of how long mass treatment could be sustained kept recurring as it was difficult to envisage an indefinite period of effective delivery. Thus the results of a study in Mali and Senegal that provided the proof of principle of elimination of onchocerciasis in Africa with ivermectin mass treatment [15] and that of a study in Kaduna, Nigeria that showed zero prevalence of infection after 17 years of ivermectin mass treatment [16], were welcome findings. With the subsequent change in 2009 in the objective of APOC from control to elimination of onchocerciasis where feasible, a new and exciting chapter for onchocerciasis in Africa was opened. With it also came many issues which needed to be addressed. These issues are, indeed, being addressed but the process is predominantly influenced by the relatively limited American experience. The enormous African experience in onchocerciasis control and elimination by the African Programmes, and the lessons learned therefrom risk being forgotten. This article discusses the key lessons learnt from the African elimination effort (summarized in Table 1) and why they are critical for successful elimination in Africa, particularly if the 2025 deadline for onchocerciasis elimination is to be achieved.

**Table 1:** Key lessons learned from onchocerciasis elimination in Africa

1. The proof of principle of onchocerciasis elimination in Africa has been established for vector control and mass treatment with ivermectin.	
2. The reproductive lifespan of the adult worm is on average 10 years. Repeated ivermectin treatment reduces adult worm lifespan and/or productivity.	
3. Long distance migration of up to 500 km, by infective vectors, can maintain transmission at the point of their arrival despite local control activities. Knowledge of local vector species is therefore important to aid in addressing the phenomenon.	
4. Community directed treatment with ivermectin is effective and sustainable.	
5. The number of years of mass ivermectin treatment required to achieve elimination is not constant but varies with endemicity level at the onset and treatment coverage. For annual treatment it ranges from 6 to 8 years for hypoendemic areas to over 20 years for holoendemic foci.	
6. Evaluation of progress towards elimination involves comparing observed and predicted infection levels after correction for endemicity and reported treatment coverage. It is essential for planning and identifying areas with insufficient progress. The latter is usually due to treatment coverage problems and its timely detection/correction is critical.	
7. The skin snip is invasive and increasingly rejected by populations, but it measures active infection which makes it an effective tool for evaluating progress. Serology is less invasive but measures past exposure making it less appropriate.	
8. Epidemiological surveys or impact assessment should prioritise high-risk areas and high-risk age groups. Sampling strategies should enable detection of residual pockets of infection.	
9. OCP and APOC have established entomological and epidemiological criteria for stopping interventions. These criteria have a clearly defined epidemiological rationale and have been operationally validated at scale. There is no such epidemiological evidence yet for serology.	
10. Model predictions and empirical evidence show that infection and transmission do not have to be zero before interventions can be stopped and that low level thresholds exist at which it is safe to stop treatment. The aim of epidemiological evaluations is not to confirm zero prevalence but that the infection level is below the threshold for safely stopping treatment.	

## Main text

### Characteristics of American and African onchocerciasis

**Fig. 2:**
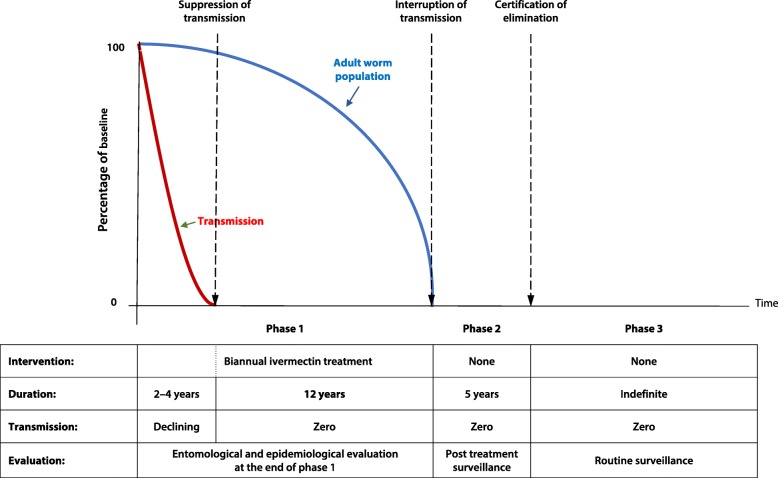
Conceptual Framework of elimination by ivermectin treatment (used by OEPA)

The American programme (OEPA) set out to eliminate onchocerciasis at its creation and pursued the objective persistently until it was achieved with remarkable success in one country after the other from 2007 to 2012 in a total of four countries [17]. The OEPA developed a strategy for pursuing interruption of transmission with ivermectin treatment which was based on studies in Guatemala [18]. The strategy consisted of treating 85% of the eligible population (equal to about 70% of the total population) with ivermectin biannually for 2–4 years to bring transmission down to zero and continuing that level of treatment to maintain zero transmission for 12 years, assuming that this would deplete the adult worm population and thus achieve elimination of transmission. Figure 2 shows the conceptual framework that was used for elimination of American onchocerciasis [19, 20]. Its logic is very similar to that of vector control by the OCP with the main difference being the required period of zero transmission, i.e. 12 versus 14 years.

Onchocerciasis in the Americas had the characteristic of being located in small foci, with a low to moderate intensity of infection and with a long history of control activities, mainly nodulectomy and vector control [11]. Vector migration was unknown and human migration did not play any significant role in spreading or even maintaining infection levels in other areas outside the foci. Furthermore, many of the vectors of onchocerciasis in the Americas are relatively inefficient compared to the vectors found all over Africa.

In a few focal areas in Africa where the endemicity of infection was similarly moderate, elimination by ivermectin treatment has also occurred, such as in the focus of Abu Hamad in Sudan using a combination of annual and biannual treatment [21], in the Kaduna focus in Nigeria using annual treatment [16], and in the river Geba valley in Guinea Bissau where elimination was already achieved in the 1990s after six years of annual ivermectin treatment only [22].

African onchocerciasis has variable epidemio-ecological settings [23–25] ranging from low and moderate intensity of infection to, and in particular, large and contiguous areas of extremely high intensity of infection maintained by highly efficient vectors. These vectors are also migratory and travel in some areas long distances of between 300 and 500 km assisted by prevailing winds [26, 27]. Millions of people are infected with many harbouring high to very high intensity of infection maintained by high vector human contact at or near vector breeding sites such as that found in the Vina valley in Cameroon and the Asubende focus in Ghana [28, 29] as well as many other holoendemic foci in Cameroon, the Democratic Republic of Congo, South Sudan and elsewhere.

### From control to elimination

Many issues need to be addressed as African National Onchocerciasis Programmes change their objectives from control to elimination. The main issues are elaborated below.

### Importance of precontrol endemicity levels

Entomological studies carried out in the course of the community trials on ivermectin demonstrated a remarkable reduction of transmission immediately following the administration of ivermectin to the population. However, unlike the studies in the Americas, the level of transmission that remained was still high. In the most thoroughly studied focus of Asubende the transmission returned to near its starting level 12 months after treatment and this finding was observed repeatedly in the first three years of ivermectin mass treatment [30]. Fitting epidemiological models to the results of these first studies provided the basis for the predictions of i) a gradual decline in transmission levels after repeated ivermectin treatment rounds, and ii) variation in the duration of ivermectin mass drug administration required to achieve elimination which ranged from 6 to more than 20 years depending on the level of endemicity at the onset of the intervention and the level of treatment coverage [10]. These predictions were later confirmed by research and evaluation data [10, 31].

**Fig. 3:**
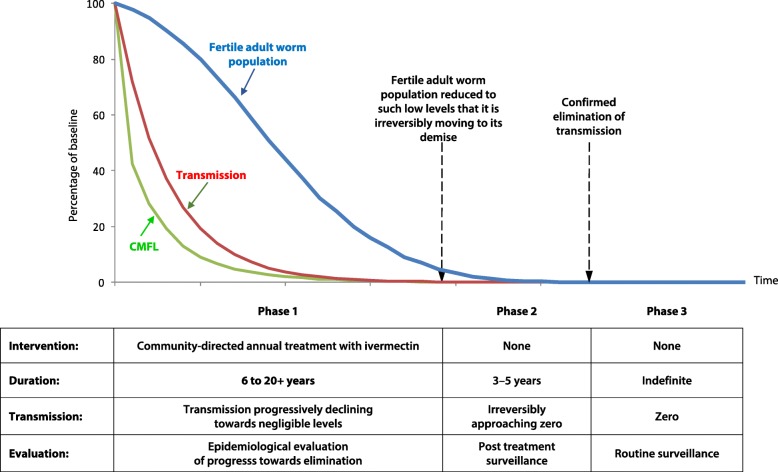
Conceptual Framework of elimination by ivermectin treatment (APOC)

Figure 3 shows the conceptual framework of onchocerciasis elimination by ivermectin mass treatment developed by APOC. It is fundamentally different from OCP’s framework for vector control which involved a rapid reduction in transmission to insignificant levels and maintaining that for 14 years till the parasite population had died out. Ivermectin treatment is less effective in reducing transmission but its comparative advantage, in addition to its microfilaricidal effect, is that it reduces the productivity and viability of the adult worms. It is the combination of these effects that determines the duration of treatment needed for elimination. In low endemic areas ivermectin treatment reduces already very low transmission to insignificant levels after only a few treatment rounds while its effect on the adult worms results in a shorter intervention period than for vector control, e.g. 6 years of annual treatment only in Rio Geba, Guinea Bissau. But in highly endemic areas longer intervention periods are needed than for vector control because of ivermectin’s more limited effect on transmission. The OEPA framework does not reflect these ivermectin dynamics but follows the vector control logic of the OCP.

### Improving and expanding treatment coverage

The intervention strategy of CDTi remains applicable during the change from control to elimination. However, the first and foremost action should be to ensure that all transmission foci that are already under treatment have and maintain high treatment coverage. Not all areas that had been identified in the era of control to undergo treatment may have had high treatment coverage [10]. It is important that areas that have not had sufficiently high treatment coverage are rapidly identified so that reasons for the poor treatment coverage can be determined and corrective measures applied to improve coverage. Experiences in APOC have shown that such detection and the application of the appropriate corrective measures can be highly effective and result in an immediate boost in coverage [10]. Equally important is ensuring 100% geographic coverage to include all endemic communities. Experience has shown that some isolated communities in less accessible areas are sometimes overlooked in treatment programmes and that these may maintain a local transmission cycle [10]. Modern mapping methods using remote sensing data and spatial models with environmental covariates such as distance to river may help refine endemicity maps and ensure that all communities which need treatment are covered [5, 32].

Next is to identify all untreated areas where there is sustained local transmission. In this regard all historical data, including that from Rapid Epidemiological Mapping of Onchocerciasis (REMO), skin snip surveys and geographic information may help identify potential transmission areas. Surveys are needed to confirm local transmission. Most of such areas will be hypoendemic areas which would not have been treated in the control period as onchocerciasis did not constitute a serious public health problem or because the REMO method with its limitation in very low endemic areas could not have properly identified them. It is also important to underline the fact that a good part of the untreated hypoendemic areas would not be independent foci. They would be tail areas of more endemic foci that have now been eliminated after 10 to 20 years treatment which has also as a consequence eliminated infection in the tail areas. The first APOC experiences with recent surveys in such areas were consistent with this hypothesis and four of the first five surveyed potential transmission areas were shown to be now skin snip negative. In general, the procedure would be to identify potential endemic areas and then carry out surveys to validate the presence or absence of infection. Isolated cases of onchocerciasis infection do not constitute evidence of local transmission. Operational research and modelling will therefore be needed to further quantify thresholds for sustained local transmission in low endemic areas where CDTi is required. The challenge will be to decide how wide to cast the net and not to start an expensive and unwarranted undertaking.

Test methods to apply should include the newly recommended tests viz. serology for detecting OV16 antibodies as well as skin snip microscopy. The attributes of both tests are already known. The serological test is more sensitive at low endemicity levels. In its Rapid Diagnostic Test (RDT) format it is easy to use, provides rapid test results and has a specificity estimated at 97–98% [33]. The ELISA version is more sensitive than the RDT but less practical for large-scale surveillance [34]. However, these serological tests cannot be used to measure active infection levels required for impact assessment and measuring progress. The skin snip microscopy has the advantage of its use for estimating active infection which is vital for measuring the progress of the intervention towards the elimination end point. It is however invasive, less sensitive in very low infections and is being increasingly rejected by the populations. The use of the two tests together, as has been done by Pauline and Surakat [35, 36], under different epidemiological and operational conditions should provide an opportunity to establish the relationship between the two tests and provide an evidence-based approach for selection of the appropriate test for different settings.

### Evaluation of progress towards elimination in all CDTi projects

Evaluation of the epidemiological impact of vector control during the OCP era was a key activity of the Programme. The process of skin snipping was applied to confirm the elimination of infection as a complement to the entomological evaluation which was applied to determine the interruption of transmission [6]. The importance of the use of two independent but complementary methods became even clearer in the OCP when the evidence of continued transmission at two foci in Burkina Faso was provided by epidemiological evaluations in the nineties, following interruption of transmission in the core area of the OCP. In the focus of Dienkoa, entomological evaluations missed a residual transmission which was detected by epidemiological evaluations. Vector control was subsequently extended to this area and effectively interrupted this local transmission. Likewise, a new breeding site with local transmission near two village settlements which had been created following the construction of a small dam on an affluent of the Bougouriba River, was not detected initially by entomological evaluations [37]. As the breeding site was therefore not covered by vector control, the resulting transmission maintained a prevalence of infection as high as 50% which, when vector control was stopped in this river basin, led to recrudescence of transmission. It was the epidemiological evaluation which brought conclusive evidence on the occurrence of the recrudescence.

With the advent of ivermectin the epidemiological evaluation process was modified accordingly in order that correct and appropriate interpretation of results would be obtained. The measure of active infection could be assessed meaningfully and comparatively only when skin snip was carried out a year after the last administration of ivermectin. The process is well established and despite all associated inconveniences, skin snip microscopy is still the epidemiologically most meaningful test that can be applied in the African setting.

In the context of elimination it is imperative to evaluate the progress towards elimination in all CDTi projects and take corrective action wherever needed. APOC has developed a methodology for the evaluation and interpretation of results which has been built on OCP’s methodology and experience. The details are provided in the publication by Tekle et al. [10] which reports on the current status of most of the CDTi projects of the former APOC. The recommended procedure is to carry out the first evaluation after six years of intervention to determine the decline in the prevalence of infection and Community Microfilarial Load (CMFL) of selected communities that may be sentinel villages or first line villages close to breeding sites, and to repeat the process every three to four years till the elimination threshold is reached. The measure can only be made with skin snip microscopy as serology cannot measure decline of infection levels. Furthermore, serology is only recommended for use in children less than ten years of age, which in onchocerciasis is the age group at lowest risk [38], whilst adults have the highest risk of infection and therefore form the most important age group for evaluation. The interpretation of the observed decline in the prevalence of microfilaria makes use of modelling to determine whether the decline is satisfactory or unsatisfactory, given the local endemicity before the intervention and treatment coverage [10]. In the event the decline is satisfactory the model is used to predict when elimination threshold will be reached. In the event of unsatisfactory decline it becomes necessary to identify the reasons in order to apply appropriate corrective measures.

Following the closure of APOC in 2015, after it had achieved its original objective, national onchocerciasis elimination committees have been established, as recommended by the WHO Guideline document of 2016 [39, 40], to coordinate the remaining activities in their countries. These committees need technical support for progress evaluation. The WHO guidelines document does not address the evaluation of progress towards elimination with ivermectin treatment nor was it its objective, and countries and partners working in the African sub-region need to urgently agree on standardised evaluation procedures and timelines.

### Are measures being currently applied sufficient to achieve elimination by 2025?

CDTi projects for which the predicted end dates of treatment are beyond 2025 may require an alternative intervention strategy to accelerate infection decline towards elimination. One option may be biannual treatment. This should however not be done indiscriminately. In areas where transmission is seasonal it will be important to determine whether there is an advantage in changing from annual to biannual mass treatment. Cost implications of such decisions should be critically considered. Model predictions and epidemiological evidence indicate that 6 to 8 years of annual treatments will be sufficient to achieve elimination in hypoendemic areas [22, 41] and changing to biannual treatment in such areas would be completely unnecessary and a waste of resources. On the other hand, holo-endemic areas, where annual ivermectin treatment has occurred over the last 15–20 years without reaching the point of stopping intervention may consider implementing biannual treatment to accelerate the attainment of the end game. However, there is no guarantee that this will achieve timely elimination as models predicted that changing from annual to biannual treatments will only reduce the remaining number of years of treatment by one third [41]. In all these cases it remains important that a high treatment coverage rate is ensured.

Many have recommended vector control as an additional intervention method to accelerate the end game. In this connection, it is worth noting that an analysis of the combined use of vector control and ivermectin mass treatment in the OCP indicated that elimination could be achieved after 12 years, only two years shorter than the duration required by vector control alone [42]. This would suggest that vector control as an additional tool would not reduce the minimum duration of the intervention below 12 years as vector control has no effect on the longevity of the adult worm.

The application of a safe macrofilaricide that can sterilise or kill the adult worm and is suitable for mass administration would still be the ideal way to accelerate the attainment of elimination of human onchocerciasis, but such a drug continues to be elusive. However there might be cases where the use of doxycycline against wolbachia may be considered [43]. This could be applied in a setting where a small proportion of highly infected people in the population continues to maintain transmission in a focus. In this connection the results of new studies on the control of wolbachia with new antibiotics will be a welcome development. A phase III trial of moxidectin has confirmed with large numbers its superior capacity, compared to ivermectin, of significant delay of microfilarial repopulation of the skin [44]. Modelling this effect suggests that moxidectin might reduce the required duration of treatment by 30 to 40%, making it more cost-effective than biannual ivermectin treatment assuming the drug would be available free of charge [45]. For the moment we can only await its registration, which should provide a welcome alternative treatment in some of the areas where it would be required to accelerate the attainment of elimination.

There are also some onchocerciasis areas co-endemic with *Loa loa* where the current intervention method with ivermectin mass treatment is not safe [46]. Most of these areas had meso and hyperendemic onchocerciasis where ivermectin treatment was justified to prevent severe complications of onchocerciasis. However, in the remaining, largely hypoendemic foci, alternative or innovative approaches need to be applied to be able to achieve elimination in the countries where this phenomenon exists. Finally, there are still areas where there is political conflict with displaced populations which impedes smooth ivermectin mass treatment, notably in South Sudan and the Democratic Republic of Congo as well as in local areas in other countries. The CDTi strategy has proven effective and robust for these problem areas but additional financial and operational support will be needed if the elimination deadline of 2025 is to be met.

Vector and human migration play a very significant role in the transmission of onchocerciasis in West Africa, particularly in the former OCP countries. It is therefore important to look out for the phenomenon and take appropriate actions. At the beginning of the rainy season long distance migrating vectors from the south travel up to 500 km in north-easterly direction, assisted by winds, to populate rivers in the middle of the OCP area. They might bring infection from their source to areas that might not be under treatment or bring new infections to areas under treatment, which could seriously complicate local intervention efforts. The reverse occurs during the dry season with long distance migration from the north to the south-west [27]. It is therefore important to coordinate treatment and in fact organise treatment in the source area just before the start of vector migration to limit the effect of the phenomenon. An inter-country cooperation should be welcome to study and mitigate the phenomenon. This long distance vector migration is one of the possible reasons for the recent occurrence of recrudescence of infection in the already controlled area in the South-West of Burkina Faso after 20 years without local transmission [47]. Dispersal of vectors from one transmission focus to another can also occur locally and delay elimination efforts. This may be especially important across national borders necessitating particular cooperation. Also important is human migration, including for example, fishermen travelling along the river from untreated to treated areas and back to their origin. Human migration to mining areas and plantations occurs all the time. It is therefore important to pay particular attention to such phenomenon and ensure that migrating people get treatment where they have arrived in the event they have not been treated already at their place of origin.

### When to stop control activities (vector control, ivermectin)

Vast experience with stopping vector control in the OCP over an area of 500 000 km^2^ demonstrated that prevalence and transmission do not have to be zero before interventions can be stopped but that low level thresholds exist when it is safe to stop intervention [13]. This process was supported by modelling and an entomological elimination threshold was given as < 0.5 infected fly per 1000 flies [48]. This threshold was subsequently also operationalised in the Americas. Follow up studies have confirmed the correctness of the OCP strategy [14, 29]. At the time of stopping vector control the average prevalence of microfilaria in the OCP was still 1.4%, consistent with modelling, and when vector control was stopped, there was no recrudescence of transmission. The study on the proof of principle on the feasibility of elimination of onchocerciasis with ivermectin mass treatment, carried out in Mali and Senegal, was also based on a stopping threshold above zero prevalence. After 15 to 17 years of annual (in two foci) and biannual (in one focus) of ivermectin treatment, the observed prevalence of infection (all ages) was 0.1–0.8% and the vector infectivity rate 0.0–0.46 infective flies per 1000. Again, when treatment was stopped, there was no recrudescence suggesting that the thresholds were valid for this epidemiological situation.

In contrast, the stopping point has not been clearly defined epidemiologically for serology. No rationale has been given for the threshold of 0.1% in children and now, as mentioned in the literature, the use of RDT is not feasible for that threshold given its specificity of 98% [35]. This anomaly is now being addressed by modelling and field studies but in the meantime the introduction of serology has delayed progress with stopping treatment which according to APOC evaluations should already be feasible for millions of people.

## Discussion

The advantages accruing from achieving elimination of a disease over simply controlling it are clear from their definitions. Control of a disease is encumbered with the continuation of the intervention activities without cessation. With elimination of a disease the intervention activities cease on attainment of elimination, giving way to surveillance activities which invariably are economically advantageous and involve significantly reduced effort. However the intervention effort required to achieve elimination is huge, exact and cannot be compromised as a rule. This is the reason why the criteria and procedures required for the intervention effort of elimination need to be clearly defined and followed precisely.

It had been quite apparent after the first years of use of ivermectin for mass treatment against onchocerciasis that elimination was likely to be achieved in the setting of low onchocerciasis endemicity whilst it appeared more problematic in the setting of high and holo-endemic onchocerciasis zones [28, 30]. It was under this premise that it was decided to do everything possible to eliminate onchocerciasis in the Americas whilst in Africa the goal was set at bringing the disease to a tolerable level from a public health point of view while collecting further information on the long-term impact of ivermectin mass treatment on onchocerciasis infection and transmission. It was thus a great relief when it was demonstrated in principle that it was also possible to eliminate onchocerciasis with ivermectin mass drug administration in hyperendemic foci in Africa [15, 16]. In effect, the empirical findings confirmed model predictions that had been based on early studies on the epidemiological impact of ivermectin. These model predictions indicate, and the empirical data confirm, that ivermectin eliminates onchocerciasis not over a fixed period of treatment but over a range of periods from 6 to over 20 years treatment depending on the level of endemicity at the onset of intervention and the coverage level of treatment of the population.

The initial presumption that ivermectin was only a microfilaricide with a limited impact on the adult female worm was the basis for the conclusion that ivermectin mass treatment would not be capable of eliminating onchocerciasis from most parts of Africa where the onchocerciasis epidemiology was complex. However, already during the clinical trials ivermectin was found to have the unusual property of suppressing the release of newly formed microfilariae from the uterus of the adult female worm [49]. A later study found that repeated treatment with ivermectin led to the attrition of female adult worms [50]. Furthermore, two independent model based analyses of available longitudinal epidemiological data from ivermectin treatment in OCP countries estimated that the productivity and/or reproductive lifespan of the adult worm is significantly reduced after repeated ivermectin treatment [51, 52]. The full effect of ivermectin on the female adult worm was not very clear at the onset of its use and it is still not exactly known how the deleterious effect of ivermectin on the adult female worm occurs. This has contributed, to some extent, to the uncertainty around the determination of the reproductive lifespan of the adult female worm with the use of ivermectin mass treatment in different epidemiological settings. There is however little doubt that areas with high endemicity of infection would require a longer duration of ivermectin mass treatment even when all persons eligible for treatment were treated. As high endemicity levels are related to the human vector contact, it can be safely assumed that the break point for interruption of transmission would differ under different epidemiological settings and indeed models predict that breakpoints in high endemicity settings would have a lower infection level threshold than breakpoints in low endemicity settings [53].

The diagnostic tool for use in onchocerciasis intervention efforts has, until recently, been the skin snip. Although standardised, reproducible, simple to use and relatively cheap, the skin snip has always had the set-backs of being invasive and low in sensitivity when the prevalence of infection is low. These set-backs, however, did not impede its effective use when local elimination was achieved over a large area in the early nineties by the OCP. OCP actually developed epidemiological indicators for stopping vector control using the skin snip method. The indicators were based on a model-based analysis of the extensive empirical data reflecting the unique epidemiology of onchocerciasis in Africa where onchocerciasis exists in large contiguous areas with an intensity of infection determined by vector human contact at the breeding site which declines the further away the human settlement is located therefrom. The OCP indicators for determining the end game of onchocerciasis using skin snip methodology were designed to target high risk age groups in areas selected by epidemiological stratification and weighting. The aim was not to detect all microfilaria positives including very low level infections. It was to determine whether the epidemiological situation was below the threshold for elimination so as to guide decision making on stopping treatment. This approach contrasts sharply with the serological approach, the basis of which has not been clearly described epidemiologically. It would appear that despite its attributes the serological test does not fit the role it is being made to fill and its introduction has given rise to confusing survey results that have complicated decision-making on stopping ivermectin treatment [33, 35, 54].

## Conclusions

Among the lessons learnt from 40 years’ experience with onchocerciasis control and elimination in Africa is that unique epidemiological differences exist between different bioecological settings that influence the effectiveness of intervention. With ivermectin mass treatment, these epidemiological differences largely determine i) the impact of ivermectin mass treatment on onchocerciasis infection and transmission and ii) the duration until treatment can be safely stopped. It is therefore important not to apply methods used in one onchocerciasis ecological setting indiscriminately to a different ecological setting; and not to ignore procedures proven to be effective in multiple settings in Africa.

New tools and methods need to be tried out for effectiveness in different epidemiological and operational settings, and compared with established procedures before application. The modality around progress evaluation and thresholds for safely stopping treatment, which have already been determined and operationalised by the African programmes, should under normal circumstances not be ignored but form important benchmarks for new tools. Such issues need to be resolved as soon as possible in order to avoid the application of inappropriate tools and methodologies which could possibly delay unnecessarily the detection of unsatisfactory progress towards elimination or the timing of stopping intervention when indeed the threshold of stopping might already have been reached. In the event, there would be a risk of missing the deadline that has been set for the achievement of elimination of onchocerciasis, and 2025 would only be a dream.

## Additional file

Additional file 1: Multilingual abstract in the five official working languages of the United Nations. (DOCX 65 kb)

## Abbreviations

APOC: African Programme for Onchocerciasis Control
CDTi: Community Directed Treatment with ivermectin
CMFL: Community Microfilarial Load
OCP: Onchocerciasis Control Programme in West Africa
OEPA: Onchocerciasis Elimination Program for the Americas
RDT: Rapid Diagnostic Test
REMO: Rapid Epidemiological Mapping of Onchocerciasis
WHO: World Health Organization
