# Supplementary material for: Is onchocerciasis elimination in Africa feasible by 2025: a perspective based on lessons learnt from the African control programmes
Source: Infect Dis Poverty. 2018 Jul 3;7:63. doi: 10.1186/s40249-018-0446-z (PMC6029117; doi:10.1186/s40249-018-0446-z)

Translation of the abstract into the five official working languages of the United Nations

**هل من الممكن القضاء على داء العمى النهري في أفريقيا بحلول عام 2025: منظور يستندُ إلى الدروس المستفادة من برامج المكافحة الأفريقية**

كاي.واي دادزي، ويو.في أمازيغو، وبي. أي بوتين وأي. شيكيتيلي

**الملخص**

**الخلفية:** يتواجد داء العمى النهري على نحوٍ سائد في أفريقيا حيثُ بدأت مكافحة نواقل المرض على نطاق واسع في عام 1974. أتاحت حملة تسجيل دواء الإيفيرمكتين والتبرع به عن طريق شركة Merck & Co في عام 1987 توفير العلاج الشامل باستخدام دواء الإيفيرمكتين في جميع البلدان الموبوءة بالمرض في أفريقيا والأمريكتين. على الرغم من أن القضاء على داء العمى النهري باستخدام دواء الإيفيرمكتين كان يُعتبر مجدياً فقط في الأمريكتين، إلا أنه وُجِدَ حديثاً أن من الممكن أن يكون مجدياً في أفريقيا كذلك، مما يستدعي تغييرات جوهرية في النهج والإجراءات الفنية والتنفيذية.

**المتن:**  لقد نجح البرنامج الأمريكي (OEPA) الذي يعمل في البيئات الموبوءة بداء العمى النهري المماثلة للحد المعتدل لسبب الانتشار المعقد لوباء العمى النهري في أفريقيا في القضاء على العمى النهري من ٤ من بين البلدان الستة الموبوءة بالمرض. وقد أُنجِزَ هذا عبر المعالجة الشاملة مرة كل عامين باستخدام دواء الإيفيرميكتين في 85% من السكان المؤهلين، والمراقبة والمتابعة باستخدام الاختبارات المصلية في الأطفال والفحوص الحشرية.

كانت نقطة بداية أول برنامج أفريقي (OCP) قبل ما يقارب العقدين من الزمان. قام البرنامج باستخدام مكافحة نواقل المرض واكتساب الكثير من المعرفة المتعلقة بديناميكيات القضاء على داء العمى النهري عبر نطاق واسع من البيئات الموبوءة بالمرض في المساحة الشاسعة لمنطقته الرئيسية. استخدم البرنامج الأفريقي OCP على نطاق واسع مؤشرات النماذج والتخلص التشغيلي للتقييم الحشري والوبائي باستخدام إجراءات عملية القصاصة الجلدية.

وقد استخدم البرنامج الأفريقي اللاحق (APOC) بشكل أساسي العلاج بالإيفرمكتين. في بادئ الأمر كان البرنامج يهدفُ إلى السيطرة على داء العمى النهري كمشكلة صحية عامة ولكن هذا الهدف تم توسيعه لاحقاً ليشمل القضاء على داء العمى النهري حيثما كان ذلك ممكناً. وبناءً على الخبرة المكتسبة من القضاء على داء العمى النهري بالبرنامج الأفريقي OCP، فقد استفاد البرنامج الأفريقي اللاحق APOC من الخبرة الكبيرة لوضع النماذج لدى البرنامج الأفريقي وقام بتطوير إجراءات تنفيذية ومؤشرات لتقييم التقدم المحرز نحو القضاء ووقف المعالجة الجماعية لداء العمى النهري باستخدام دواءالإيفيرمكتين في البيئة الأفريقية المعقدة.

**الخُلاصة:** عقب إغلاق البرنامج الأفريقي اللاحق في عام 2015، يبدو أن تنفيذ عملية القضاء على داء العمى النهري في أفريقيا يغفل جميع الخبرات التي قد تراكمت من البرامج الأفريقية. فهو يستخدم بشكل رئيسي العمليات الأمريكية التي وُضعت في بيئة غير مماثلة للبيئة الأفريقية المعقدة التي ينتشر بها داء العمى النهري. وهذا يعوق التقدم نحو اتخاذ قررات لوقف التدخل في العديد من المناطق التي قد بلغت نقطة القضاء على الداء. تُلخصُ هذه المقالة الدروس المستفادة في أفريقيا وأهميتها في تحقيق القضاء على الداء في أفريقيا بحلول عام 2025.

Translated from English version into Arabic by [Bashaier Allam](http://twb.translationcenter.org/workspace/accounts/view/id/53780) and free bird, through


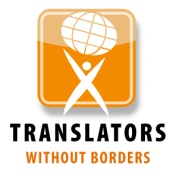


**基于非洲控制项目经验评估2025年非洲消除盘尾丝虫病的可行性**

K.Y. Dadzie, U.V. Amazigo, B.A. Boatin and A. Sékétéli

**摘要**

**引言：**非洲是盘尾丝虫病的主要流行区，1974年起开始大规模病媒控制。1987年，默克公司完成了伊维菌素的注册和捐赠，该药使非洲和美洲的盘尾丝虫病流行国家得以开展大规模治疗。虽然目前仅在美洲国家证实了利用伊维菌素消除盘尾丝虫病的可行性，但是近年来，在非洲国家使用伊维菌素消除盘尾丝虫病也显现出一定的可行性，因此技术和操作方法以及程序需要进行重要改进。

**正文：**在类似于非洲盘尾丝虫病复杂流行病学情况的轻度状态下，美国项目(OEPA)成功地在4个（4/6）流行国家中消除盘尾丝虫病。该项目在符合条件的人群中（85%）开展伊维菌素群体治疗（每年两次），并使用儿童血清学与昆虫学检查进行监测评估。

第一个非洲项目（OCP）已开展近20年。在其广阔的核心区域内，该项目它在广泛的流行区中大范围地开展媒介控制，积累了大量有关盘尾丝虫病消除动态的知识。OCP广泛利用建模和量化消除指标开展昆虫学评估，运用皮片检测法进行流行病学评价。

后续非洲项目(APOC)主要开展伊维菌素治疗。初步目标是把盘尾丝虫病作为一种公共卫生问题加以控制，但这一目标后经扩展，纳入在可行区域里消除盘尾丝虫病。基于OCP消除盘尾丝虫病的经验，APOC借鉴OCP丰富的建模经验，开发了操作程序和指标以评估在非洲复杂背景下的盘尾丝虫病消除进展并阻止使用伊维菌素进行群体治疗。

**结论：**2015年APOC结束后，非洲盘尾丝虫病消除行动似乎忽略了非洲项目积累的所有经验。它主要采用美国方案，但这些方案是基于不同于非洲复杂的盘尾丝虫病情况的环境中制定的。这是阻碍了决策进展，已达到消除点很多地区停止干预。本文总结了非洲盘尾丝虫病消除经验及其在2025年之前实现消除的重要性。


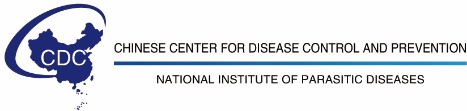
Translated from English version into Chinese by Fan Yang, edited by Jin Chen

**Pourra-t-on éliminer l’onchocercose en Afrique d’ici 2025 ? Point de vue basé sur les enseignements tirés des programmes africains de lutte contre la maladie.**

KY Dadzie, UV Amazigo, BA Boatin et A. Sékétéli

**Résumé**

**Contexte :** L’onchocercose sévit principalement en Afrique où une lutte antivectorielle à grande échelle a débuté en 1974. L’autorisation de mise sur le marché de l’ivermectine en 1987 et des dons de ce médicament par le laboratoire Merck & Co ont permis d’administrer un traitement de masse dans tous les pays d’endémie en Afrique et en Amérique. Bien que l’élimination de l’onchocercose grâce à l’ivermectine n’ait été jugée possible qu’en Amérique, on estime depuis peu qu’elle le serait aussi en Afrique, à condition d’opérer des changements fondamentaux dans les méthodes et procédures techniques et opérationnelles.

**Corps du texte :** Le programme américain de lutte contre l’onchocercose (OEPA), déployé des contextes épidémiologiques proches des situations les moins graves du spectre épidémiologique complexe de l’Afrique, est parvenu à éliminer l’onchocercose dans 4 des 6 pays d’endémie. Cela a été rendu possible par un traitement de masse bisannuel, administré à 85 % de la population éligible, et à un suivi et une évaluation utilisant des tests sérologiques chez les enfants et des tests entomologiques.

Le premier programme africain (OCP) avait près de vingt ans d’avance sur son homologue américain. Basé sur la lutte antivectorielle, il a permis d’acquérir des connaissances considérables sur la dynamique de l’élimination de l’onchocercose dans des contextes épidémiologiques très divers, au cœur de son vaste foyer principal. L’OCP a eu largement recours à la modélisation et à des opérateurs d’élimination opérationnels pour l’évaluation entomologique et épidémiologique par biopsies cutanées.

Le programme africain qui lui a succédé (APOC) s’appuyait principalement sur le traitement à l’ivermectine. Initialement conçu pour lutter contre l’onchocercose en tant que problème de santé publique, il a été élargi à l’élimination de l’onchocercose là où cela était possible. S’appuyant sur l’expérience de l’OCP en matière d’élimination de l’onchocercose, et de la modélisation épidémiologique, l’APOC a élaboré des procédures opérationnelles et des indicateurs permettant d’évaluer les progrès dans l’élimination de la maladie et à mettre fin aux traitements de masse dans le contexte complexe du continent africain.

**Conclusion :** Depuis la fin de l’APOC en 2015, les actions visant à éliminer l’onchocercose en Afrique semblent négliger toute l’expérience acquise par les programmes africains. Elles utilisent principalement des méthodes développées en Amérique, dans un environnement différent de l’épidémiologie complexe de l’onchocercose en Afrique. Il devient ainsi difficile de décider de l’arrêt des interventions dans de nombreuses régions qui ont atteint le point d’élimination. Cet article résume les enseignements de l’expérience africaine et leur importance pour parvenir à éliminer la maladie en Afrique d’ici 2025.

Translated from English version into French by Céline Cloarec and Suzanne Assenat, through


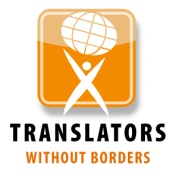


**Возможна ли ликвидация онхоцеркоза в Африке к 2025 году? Обзор опыта, извлеченного из африканских программ по борьбе с онхоцеркозом**

К. И. Дадзи, У. В. Амазиго, Б. А. Боатин и А. Секетели

**Аннотация**

**Контекст исследования:** Онхоцеркоз встречается преимущественно в Африке, и в 1974 году на континенте начали крупномасштабную борьбу с переносчиками этого заболевания. Благодаря регистрации и пожертвованию ивермектина компанией Merck & Co в 1987 году появилась возможность массового лечения этим препаратом во всех эндемичных странах Африки, а также в Северной и Южной Америке. Ранее лечение онхоцеркоза ивермектином считалось осуществимым только в Северной и Южной Америке, однако недавно было показано, что это возможно и в Африке. Следовательно, возникла необходимость фундаментальных изменений как в технических, так и в оперативных подходах и процедурах.

**Основная часть:** Американская программа (OEPA), которая применяется в эпидемиологических условиях онхоцеркоза, сходных с легкой формой комплексной эпидемиологии этой инфекции в Африке, успешно ликвидировала онхоцеркоз в 4 из 6 его эндемичных стран. Это достижение стало возможным благодаря массовому лечению ивермектином 85% больных два раза в год, а также благодаря мониторингу и обследованию с применением серологических тестов у детей и энтомологических тестов у взрослых.

Первая африканская программа (OCP) началась почти два десятилетия назад. В рамках программы велась борьба с переносчиками заболевания, и в ходе ее осуществления был накоплен большой объем знаний о динамике ликвидации онхоцеркоза в самых разных эпидемиологических условиях во основной области распространения инфекции. OCP широко применяла моделирующие и операционные индикаторы ликвидации для энтомологической и эпидемиологической оценки с помощью кожных надрезов.

Вторая африканская программа (APOC) в основном затрагивала лечение ивермектином. Первоначально ее целью являлось сдерживание онхоцеркоза как проблемы общественного здравоохранения, но позднее эта цель была расширена до ликвидации этого заболевания везде, где это возможно. На основе опыта ликвидации онхоцеркоза, а также богатого опыта моделирования в рамках программы OCP, APOC разработала оперативные процедуры и показатели для оценки темпов ликвидации онхоцеркоза и прекращения массового лечения ивермектином в сложных условиях Африки.

**Заключение:** После закрытия APOC в 2015 году процесс ликвидации онхоцеркоза в Африке, по-видимому, не учитывает весь опыт, накопленный африканскими программами. Сегодня для ликвидации онхоцеркоза применяются преимущественно американские методы, которые были разработаны в условиях, отличающихся от сложных условий онхоцеркоза в Африке. Это затрудняет процесс принятия решений по прекращению вмешательства в те области, где заболевание уже ликвидировано. В данной статье обобщаются уроки, извлеченные в Африке, а также подчеркивается их важность для того, чтобы добиться ликвидации онхоцеркоза в Африке к 2025 году.

Translated from English version into Russian by Polina Nikitina and Tatiana Kary, through


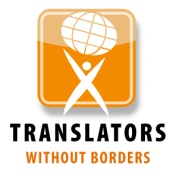


**¿Es factible la eliminación de la oncocercosis en África para el 2025?: Una perspectiva basada en lo que aprendimos gracias a los programas de control en África.**

KY Dadzie, UV Amazigo, BA Boatin y A. Sékétéli

**Resumen**

**Contexto:** La oncocercosis se encuentra sobre todo en África, en donde en 1974 comenzó el control a gran escala del vector que la ocasiona. En 1987, el registro y la donación de ivermectina por Merck & Co permitieron el tratamiento masivo en todos los países endémicos de África y las Américas. Si bien se consideraba que la eliminación de la oncocercosis con ivermectina solo era viable en las Américas, recientemente se ha demostrado que, con cambios fundamentales en los enfoques y procedimientos técnicos y operativos, es posible eliminarla también en África.

**Cuerpo principal:** El programa para la eliminación de la oncocercosis en las Américas (OEPA, por sus siglas en inglés), que opera en escenarios epidemiológicos similares al extremo moderado de la compleja epidemiología de la oncocercosis en África, ha logrado eliminar la oncocercosis en 4 de sus 6 países endémicos. Esto se logró mediante el tratamiento masivo bianual con ivermectina en el 85% de la población elegible, el seguimiento y la evaluación con pruebas serológicas en niños y las pruebas entomológicas.

El primer programa africano (OCP, por sus siglas en inglés) tuvo casi dos décadas de ventaja. Utilizó el control de vectores y acumuló una gran cantidad de información acerca de la dinámica de la eliminación de la oncocercosis en un amplio rango de entornos epidemiológicos en la gran extensión de su zona central. El OCP hizo un uso extensivo de modelos y de indicadores operacionalizados de eliminación para la evaluación entomológica, y de procedimientos de biopsia cutánea (en inglés "skin snip") para la evaluación epidemiológica.

Lo siguió el Programa africano para el control de oncocercosis (APOC, por sus siglas en inglés), que aplicó sobretodo el tratamiento con ivermectina. Su objetivo inicial era controlar la oncocercosis como problema de salud pública, pero ese objetivo se amplió más tarde para incluir la eliminación de la oncocercosis en donde fuera posible. Basándose en la experiencia de la OCP en la eliminación de la oncocercosis y en la elaboración de modelos, la APOC ha desarrollado procedimientos operacionales e indicadores para evaluar el avance en la eliminación, y ha detenido el tratamiento masivo de la concocercosis con ivermectina en el complejo entorno africano.

**Conclusión:** Después del cierre de APOC en el año 2015, la puesta en marcha de la eliminación de la oncocercosis en África parece dejar de lado toda la experiencia adquirida gracias a los programas africanos. Utiliza procesos en su mayoría americanos, que fueron desarrollados en un entorno diferente al del complejo escenario africano. Esto impide avanzar hacia la adopción de medidas que detengan la intervención en muchas áreas que han alcanzado ya el punto de eliminación. Este artículo resume las lecciones aprendidas en África y su importancia para lograr eliminar la oncocercosis en África para el año 2025.

Translated from English version into Spanish by [Lidia Norese](http://twb.translationcenter.org/workspace/accounts/view/id/55859) and [Maria CG](http://twb.translationcenter.org/workspace/accounts/view/id/55775), through


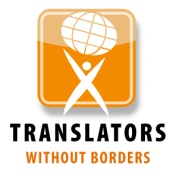

Supplement: Supplementary file 1 — Multilingual abstract in the five official working languages of the United Nations. (DOCX 65 kb) [file 40249_2018_446_MOESM1_ESM.docx]
